# Occurrence of Chemical Contaminants in Peruvian Produce: A Food-Safety Perspective

**DOI:** 10.3390/foods10071461

**Published:** 2021-06-24

**Authors:** Oscar A. Galagarza, Alejandra Ramirez-Hernandez, Haley F. Oliver, Mariel V. Álvarez Rodríguez, María del Carmen Valdez Ortiz, Erika Pachari Vera, Yakelin Cereceda, Yemina K. Diaz-Valencia, Amanda J. Deering

**Affiliations:** 1 Department of Food Science, Purdue University, 745 Agriculture Mall Drive, West Lafayette, IN 47907, USA; alejaramirez02@gmail.com (A.R.-H.); hfoliver@purdue.edu (H.F.O.); adeering@purdue.edu (A.J.D.); 2 Academic Department of Process Engineering, Universidad Nacional de San Agustin, Arequipa 04001, Peru; malvarezro@unsa.edu.pe (M.V.Á.R.); epachari@unsa.edu.pe (E.P.V.); ydiazva@unsa.edu.pe (Y.K.D.-V.); 3 Department of Biological Sciences, Universidad Nacional de San Agustin, Arequipa 04001, Peru; mvaldezor@unsa.edu.pe; 4 Department of Sociology, Universidad Nacional de San Agustin, Arequipa 04001, Peru; ycereceda@unsa.edu.pe

**Keywords:** Peruvian agriculture, food safety, public health, pesticide residues, heavy metals, mycotoxins

## Abstract

The presence of chemical contaminants in agricultural products is a continued food-safety challenge in Peru. This country has robust agriculture potential, but its output of fruits and vegetables is severely impacted by massive mining activities, as well as poor farming practices, including the use of polluted irrigation water, misuse of pesticides, and inadequate postharvest conditions. This review examines the current scientific knowledge on the levels of pesticide residues, heavy metals, and mycotoxins on crops produced in Peru. The available data shows that several crop varieties are contaminated with these classes of chemical contaminants, and at levels that exceed the national and international permissible limits. The abundance of chemical contaminants in produce indicates a relevant food-safety issue, which increases the risks of chronic human diseases, like cancer—a leading cause of death in Peru. Finally, this review presents recommendations to address these contamination problems in produce grown in the Andean country.

## 1. Introduction

Peru is a developing economy with a massive agricultural output, which has contributed to the country’s socioeconomic prosperity. Production from the agriculture sector has a net worth of USD 5.5 billion, roughly a 7% share of Peru’s gross domestic product (GDP), and supports 30% of the country’s labor force [1,2]. Vegetables, fruits, and grains are important agricultural crops, as they are key components of the Peruvian diet and produce exports. Trade of agricultural products is vital for the country’s economy, remaining only behind mining. Traditional products (e.g., sugar and coffee) represent the dominant crop varieties exported, but the exportation of nontraditional products (e.g., asparagus, mango, avocado, and grapes) has increased dramatically in the past two decades. Agricultural exports contributed USD 3.1 billion in 2019, and the top international markets included the United States, the European Union, China, Chile, Colombia, and Mexico [1]. These increases in production and exports of fruits and vegetables are due to global consumer trends shifting toward health and wellness [3].

Despite the success of agricultural economy in Peru, intensification of agricultural production has come with important challenges. Prominent issues include lack of infrastructure investment, inadequate financial resources, limited agriculture technical knowledge, water scarcity, and poor water quality [4]. Moreover, these challenges can increase the likelihood of chemical contaminants in agricultural products, creating one major contemporary food-safety issue. The abundance of these pollutants can be difficult to remediate, given the country’s limited access to up-to-date quality data on environmental resources (e.g., irrigation water), and the absence of data attributing domestic foodborne illnesses to chemically contaminated foods.

Foodborne illnesses are an important public-health problem in Peru. Roughly 6098 people fall ill from contaminated foods each year [5]. The occurrence of foodborne diseases is not only vital from the human-welfare standpoint, but also because approximately USD 500 million is lost annually in productivity and healthcare expenses [6]. Additionally, Peru is one of the countries in the Americas that is categorized under the World Health Organization stratum “D”, which indicates a high child and adult mortality from foodborne disease [7].

Although foodborne outbreaks are more frequently due to contamination with pathogenic microorganisms, chemical compounds are also common contaminants of fresh produce. It is estimated that one to two foodborne outbreaks are attributed to chemical pollutants each year, yet precise numbers of affected individuals are not available [5]. Chemical contaminants are either of natural or anthropogenic origin, and are potentially toxic when the polluted crops are consumed by people. Problematic chemicals that frequently contaminate agricultural crops include heavy metals, mycotoxins, and pesticide residues. The health effects of these chemical contaminants can range from acute, such as organ failure, to chronic effects, such as the increased risk of cancer, and neurological disorders [8,9,10,11].

There is no safe dose for chemical contaminants, but there are acceptable doses that have been estimated to not elicit signs of toxicity. These acceptable doses are known as maximum limit, and serve as a general guideline for many contaminants across various crop varieties [12]. Compliance with this guideline ensures the harmonization of food safety and trade. Thus, monitoring systems are often established in different countries to ensure that chemical contaminants are below the maximum limits. The weak enforcement in food-safety monitoring, surveillance, and control of such chemical contaminants in Peru makes consumers potentially more vulnerable [13]. More importantly, children, old adults, and immunocompromised people are at a great risk of foodborne illness [14].

Over the last two decades, the number of studies reporting chemical contaminants in Peruvian agricultural crops has been increasing. This is an indication that food-safety concerns are gaining more attention. To the best of our knowledge, however, there is no comprehensive review on chemical contaminants covering the available data on pesticide residues, heavy metals, and mycotoxins in crop varieties cultivated in Peru. This review is a landscape analysis of scientific articles, academic theses, and Peruvian governmental agency reports for the years 2000–2021 that present data on heavy metals, mycotoxins, and pesticide residues, to depict the current safety status of produce grown in Peru. The information presented in this review will enable growers, stakeholders, policymakers, and consumers to have a more comprehensive understanding of current food-safety issues of produce grown in Peru, and advocate for the appropriate changes to current practices.

## 2. Peru Food Laws and Regulations for Chemical Contaminants

Peru has a well-established food-safety system, which is led by the General Directorate of Environmental Health and Food Safety (Dirección General de Salud Ambiental, DIGESA) and the National Service of Agrarian Health (Servicio Nacional de Salud Agraria, SENASA) agencies; the former agency is in charge of monitoring processed foods and beverages, while the latter surveils animal and plant health. Thus, the role of DIGESA is similar to that of the US Food Drug and Administration (FDA), and SENASA is the equivalent of the US Department of Agriculture’s Animal and Plant Health Inspection Service (USDA-APHIS) and Food Safety Inspection Service (USDA-FSIS).

Food-safety laws in Peru were established under the legislative decree N°1062. Decree N°004-2011-AG provides the food-safety guidelines of agrarian products for domestic and international consumption [15]. Some of these reference guidelines are presented in Table 1. These Peruvian food safety laws follow the Codex Alimentarius standards as their guidelines to promote food safety [16]. The Codex Alimentarius consists of a collection of standards and practices to ensure that foods are safe for both consumption and international trade; it was established by the Food and Agriculture Organization of the United Nations and the World Health Organization [12].

Other relevant regulations that are linked to produce food-safety laws include legislation that underlines national standards for quality of irrigation water. Such standards were established under the supreme decree N°015-2015-MINAM by the Ministry of the Environment (Ministerio del Ambiente, MINAM) in collaboration with the National Authority of Water (Autoridad Nacional del Agua, ANA), which follows recommendations by the US Environmental Protection Agency (EPA) and the WHO [17,18]. Some of the standards established for heavy metals in irrigation water are included in Table 1.

**Figure 1:**
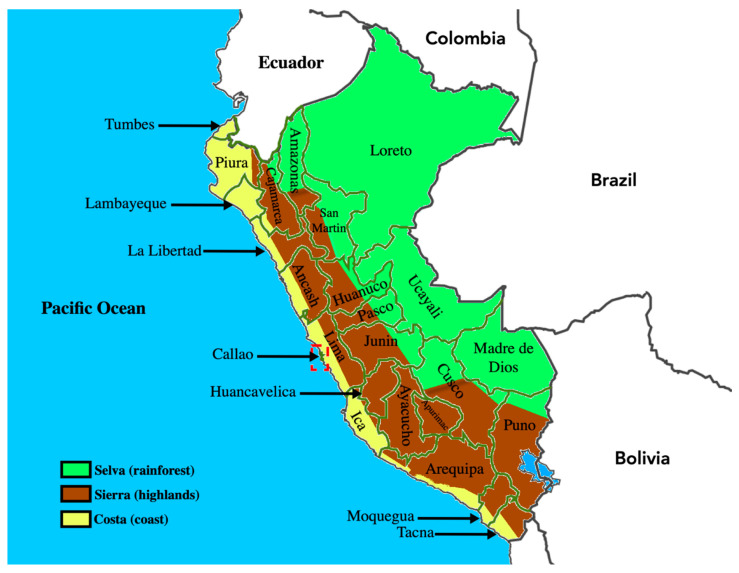
Map of Peru, showing its three geographical regions and 25 departments. ArcGIS 10.8.1 software (http://www.esri.com/software/arcgis, accessed on 5 May 2021) was used to develop the map.

Compliance with international trade laws is crucial for Peruvian producers who desire to export their crop output. Given that the US and countries in the European Union (EU) are major importers of Peruvian foods, the latter have to meet the respective food-safety requirements. The Food Safety Modernization Act (FSMA), the major food-safety standards in the US, plays a key role in import acceptance in the ports of entry. One FSMA rule in the Foreign Supplier Verification Program requires importers to provide a phytosanitary certificate, issued by Peru’s SENASA, that verifies that the product meets all the US food-safety standards [19,20]. A similar rigorous process occurs when exporting to the EU, as the legislation requires that the exporter be certified by SENASA [21]. The ability to comply with export regulations has made the food sector more vibrant, and there has been an improvement in agricultural education and infrastructure primarily [22]. However, this is an ongoing effort to lower the discrepancies in access to these benefits across all the geographical regions of Peru (refer to Figure 1 for a map of Peru); from the Costa (coast) with more mechanized agriculture, to the more small-scale agriculture that predominates in both the Selva (Amazon rainforest) and the Sierra (highlands) [23].

## 3. Inclusion Criteria for Review

The studies that were included in this comprehensive review consisted of scientific peer-reviewed works and governmental reports from Peru that focused on quantifying pesticide residues, heavy metals, and mycotoxins in produce grown in Peru. Scientific articles on heavy-metal contaminants in rivers in Peru were also included, given their potential as a source of heavy metals that can contaminate produce. The works that were considered employed analytical chemistry methods (e.g., gas or liquid chromatography and mass spectrometry) for evaluation of chemical contaminants. Relevant studies presented in academic theses were also included for this systematic review.

## 4. Chemical Contaminants

### 4.1. Pesticide Residues in Produce

Pesticides are broadly defined as plant protection products that eliminate unwanted pests, and are classified by the target pest (e.g., insecticides, herbicides, fungicides). These chemical substances can also be classified as natural or synthetic, with the latter being the most commonly used in farms. The use of pesticides in agriculture has contributed to reducing crop losses, and maintaining a steady yield of the product. Additionally, pesticides have been utilized in human medicine in the control of disease vectors (e.g., the Anopheles mosquito related to malaria). Although their utilization has been with the intention of promoting crop protection and productivity, their consistent use is known to affect human health.

The use of certain pesticides has been prohibited in Peru, and most other countries, because of their well-known potential adverse effects. Public awareness of pesticide toxicity was not achieved until the publication of the book *Silent Spring* by Rachel Carson in 1962. This book related the potential impacts of dichlorodiphenyltrichloroethane (DDT), an organochloride insecticide, on the environment, plants, animals, and human health. *Silent Spring* helped to create an environmental movement that led to the ban of DDT for agricultural purposes in the US and other nations. Peru, like other nations, has prohibited the use of organochloride insecticides (see Table S1) such as DDT since 1991 [24].

While the use of this pesticide class is illegal, there are other classes of harmful pesticides commonly used to date in Peru. Some examples include insecticides (e.g., organophosphates, carbamates, and pyrethroids), herbicides (e.g., paraquat), and fungicides (see Table S2). The Ministry of Agriculture and Irrigation (Ministerio de Agricultura y Riego, MINAGRI) reported an increase of 2.6 to 3.3 metric tons of imported pesticides to Peru from 2012 to 2016 [25]. This data is concerning, as agriculture is an important consumer sector of pesticides in Peru. Additionally, data on pesticide use suggests that increased productivity in the Costa region goes hand-in-hand with the higher use of pesticides in this area when compared with the other two regions (Table 2) [26]. The increases in pesticide application to boost production and meet consumer demands can result in a higher accumulation of the pesticide, or derivatives (termed “pesticide residues”), as the contaminated produce goes up the food chain.

Few research studies have evaluated pesticide residues in Peruvian produce. Two studies showed that the use of the organophosphate methamidophos resulted in pesticide residues in tomatoes and potatoes available in different markets in Lima [27,28]. The studies found that 9/20 (45%) sampled potatoes and 1/25 (4%) sampled tomatoes exceeded the maximum residue limit (MRL) for methamidophos (0.05 mg/kg) in that commodity (Table 3). In Peru, the MRLs of pesticides residues were established under the legislation N°1006-2016/MINSA, and when values for a given specific pesticide are not defined, the Codex Alimentarius is used as reference [29].

Governmental authorities in Peru have reported chemical contamination of produce. The SENASA agency reported the presence of pesticide residues and mycotoxins in various fruits and vegetables obtained from markets of multiple departments across Peru (Table 4). The SENASA reports from 2011–2019 show that the number of samples that exceeded the MRL for pesticide residues was ≥10% per year [31,32,33,34,35,36,37,38,39]. The majority of noncompliant samples were associated with values above the MRL of chlorpyrifos (0.05–20 mg/kg), cypermethrin (0.05–20 mg/kg), methamidophos (0.01–1 mg/kg), and tebuconazole (0.05–10 mg/kg) in every report since 2012. Tacna and La Libertad were the departments that were more frequently linked to noncompliant samples. These SENASA reports are the most comprehensive reports on pesticide-residue contamination within Peru, and offer insight that the previously mentioned pesticides tend to be problematic in produce.

There has also been Peruvian produce found to have breached the MRL of pesticides in the receiving country. The crops included lettuce, paprika, quinoa, peas, and mandarins [40] These findings were further confirmed by reports that described multiple agricultural Peruvian imports into the US that were detained due to contamination with pesticide residues (e.g., chlorpyrifos and methamidophos), or mycotoxin contamination [41]. A European study focused on fruits and vegetables from different South American countries commercially available in various Nordic countries also reported contaminated produce [30]; among the produce varieties sampled from Peru, 4/46 (8.7%) exceeded the MRL for two fungicides and one carbamate insecticide (Table 3). Even though Peruvian products had a low sample size, it is concerning that the percentage of positive samples exceeding the MRL for the pesticides was comparable to that of Colombia (*n* = 81, 10% positive) or Brazil (*n* = 233, 13% positive), which both had a larger sample size.

The inappropriate use of pesticides is a likely cause of their increased levels in produce. A study in China found that if the fungicide chlorothalonil was applied on cabbage based on their recommended frequency preharvest, the residual amount did not exceed the MRL (1 mg/kg) [42]. Another study in Chile found that 32/118 (27%) of sampled leafy greens exceeded the domestic MRL for cypermethrin (0.70 mg/kg), chlorothalonil (0.01 mg/kg), chlorpyrifos (0.05 mg/kg), and methamidophos (0.01 mg/kg) [43]. The authors of the Chilean study attributed these findings to the high frequency of applications in North Central agricultural farms of Chile. These comparisons offer a plausible explanation to the recurrent MRL breaches of certain pesticides (e.g., chlorpyrifos, methamidophos) in Peruvian crops.

Although most reports on pesticide residues are focused on produce for export purposes, there are scant studies on other crop varieties consumed internally. This excludes crop varieties such as blueberries, lucuma, leafy greens, sprouts, and quinoa, which are common components of the Peruvian diet. It could be argued that since there is no governmental push to monitor the previously mentioned crops, then growers have no increased incentive to implement better agricultural practices. The absence of data from many crops for internal consumption sheds light on a food-safety loophole, which can potentially affect their consumers.

### 4.2. Heavy Metals in Produce

Heavy metals are hazardous contaminants in living ecosystems. Some characteristics of these trace elements that make them challenging to deal with include their low biodegradability, long persistence in the environment, and ability to accumulate in living beings and subsequently cause a negative effect in the host. Their presence in produce is generally attributed to the entry of heavy metals either from natural mineral sources or anthropogenic activities (pesticides, contaminated irrigation water) into environmental matrices (soil, water, and air), which are in direct contact with plants [44]. Plants are limited in their mechanisms of nutrient absorption and cannot distinguish between the nutrients being absorbed [45]. Therefore, the degree of pollution in the environment can dictate the likelihood of plant–heavy-metal interactions. As a consequence of these interactions, the consumption of the harvested crop is a major risk of human exposure to heavy metals [46].

The rapid industrialization and a growing population contribute to heavy-metal pollution in Peru. The expansion of mining, an activity that accounts for 14% of Peru’s GDP and about 60% of exports, is an important source of heavy-metal contamination [47]. Mining practices release over 13 billion m^3^ of effluents annually, creating undesirable stress in rivers and other sources of water that are used for crop irrigation [48]. These practices result in contamination of soil and water with arsenic (As), cadmium (Cd), copper (Cu), lead (Pb), and/or zinc (Zn)—heavy metals that are often found in increased amounts in produce [49].

It has been estimated that about 1.6 million people (5% of Peru’s population) live within 5 km of active or historical mining sites, and human activity in proximity to Peruvian mines is primarily agricultural [50]. Thus, the high risk of pollution by mining effluents threatens the food safety of nearby crops. For example, one European risk-assessment study on daily heavy-metal intake in drinking water and foods found that As and Pb levels were elevated in the diets of the population that was closer to a gold mine in Cajamarca, Peru [51]. Additionally, greater use of agrochemicals (pesticides and fertilizers), or higher gas emissions from the use of leaded gasoline, may increase the levels of these trace elements in produce [52,53].

Some primary studies quantified heavy-metal contamination in produce in Peru, and these are summarized in Table 5. A group from the National University of Piura conducted a study to measure the levels of several heavy metals in 10 different vegetable varieties in the city of Piura [54]. Results from this study showed that all the evaluated vegetables registered values exceeding the maximum contaminant limit (MCL) for Pb (0.1 mg/kg). The levels of As were also noncompliant in cilantro and lettuce (>0.1 mg/kg). The work focused on produce grown in the city of Trujillo, which has the greatest production of vegetables in the north of Peru. Similarly, commercially available lettuce has also been found to exceed the MCL for Pb at 0.3 mg/kg [55].

Mining activities can influence the presence of heavy metals in agricultural crops. A study in Cajamarca showed that all sampled potatoes (*n* = 40) were contaminated with Cd (mean value of 0.31 mg/kg), exceeding its MCL at 0.1 mg/kg [67] The finding was meaningful, as the potatoes were grown in proximity to the Yanacocha mine, South America’s largest gold mine. This mine influences two of the rivers, the water of which was used to irrigate the potato crops. Another study found potatoes in Junin showed levels of Pb at 0.41 mg/kg (MCL at 0.1 mg/kg) [68] The authors explained that mining was a likely source of this contamination. Similarly, other studies in Chile, China, and Slovakia reported that produce grown near a mining operation had noncompliant concentrations of As, Pb, and Cd [70,71,72]. These findings supported the importance of understanding the risk of contamination of produce grown close to mining activities.

These studies, although useful in bringing more attention to the issue of heavy metals, do not describe the complete status of heavy-metal contamination in produce throughout Peru. In an effort to address this knowledge gap, SENASA began to include analyses of As, Cd, and Pb in produce in its annual reports in 2019 [39,73]. This may indicate a trend toward increased governmental awareness with regard to the potential chemical contaminants to which produce is exposed in Peru. However, there is also room for improvement, especially in transparency, as the 2019 report did not differentiate the contribution of specific chemical classes in noncompliant samples [39].

### 4.3. Mycotoxin Contamination in Produce

Mycotoxins are naturally occurring toxic secondary metabolites produced by fungi (e.g., Aspergillus, Fusarium and Penicillium), and are a common contaminant in food commodities around the world. Approximately 300 mycotoxins have been characterized, but the more common food contaminants include aflatoxins (AT), ochratoxins (OT), patulin (PT), and fumonisins (FM) [74]. Mycotoxin contamination can occur throughout the entire production process, from pre- to postharvest. Warm temperatures (20–28 °C) and high humidity are conditions that tend to favor the growth of mesophilic mold, resulting in greater toxin production [75]. Thus, poor drying and inappropriate storage conditions during postharvest activities will increase the risk of mycotoxin contamination.

There are a few studies that evaluated mycotoxin contamination in produce. An early study detected OT A at a mean level of 37.1 µg/kg in paprika (*n* = 130), and exceeding its MCL at 15 µg/kg [75]. Similarly, another study found that red chili peppers from Cusco were contaminated with both AT B1 and OT A [76]. This study showed that the mean concentration of OT A (29 µg/kg) was also above the MCL for OT A (15 µg/kg). Another project in the same crop reported that AF B1 contaminated 2/4 (50%) of the samples, yet at levels below the MCL established by the Codex Alimentarius at 10 µg/kg for aflatoxins [77]. A recent study reported that Peruvian purple maize (*n* = 82) was commonly contaminated with both AT B1 and FM (B1 + B2), with mean values of 2.1 µg/kg and 2.6 mg/kg, respectively [78]. While the levels of either AT and FM did not exceed the MCL established by the Codex Alimentarius, the levels of FM were above the MCL set by the EU at 1 mg/kg. The findings on FM indicated an area for improvement if Peru wants to potentiate its exports of maize to the EU. A similar study in Brazil found that 14/148 maize samples had a noncompliant concentration for FM (MCL set at 5 mg/kg in that country) [79]. The importance of these findings is related to chili peppers and corn maize being integral components in the Peruvian diet, and that also one of the major problems during the production of these crops is mycotoxin contamination [80].

The byproducts of produce are also at risk of mycotoxin contamination. For example, one Peruvian detected PT in 2/6 (33%) samples of the commercially available apple juice in Lima, using thin-layer chromatography [81]. This same work also detected the same toxin in 3/12 (25%) visibly damaged apples sampled from the market. The authors corroborated these findings by confirming the presence of Penicillium molds, a common producer of PT, in the apples. Since putrid apples may be used for juice or cider production, the Food and Agriculture Organization recommends that unfit apples are removed to avoid the likelihood of PT in apple byproducts [82].

The SENASA agency has also reported the levels of mycotoxins in two crop varieties (Table 4). The conducted analytes include total AT (B1, B2, G1, G2) and OT A only in paprika and Brazilian nuts. The number of noncompliant samples decreased from 33.3% (14/42) in 2011 to 2.53% (2/79) in 2018, which was an indication of improvements in the storage conditions of these commodities over time. However, these analyses of mycotoxins led by the government should be more inclusive of other crops, since paprika and Brazil nuts are not the only crops at risk of contamination with mycotoxins, and should not exclude other economically important crops like corn and beans [83,84].

## 5. Water and Chemical Contaminants

The water that is utilized for irrigation is a potential source of chemical pollutants. Runoffs of the mining and agriculture sectors are responsible for heavy-metal and pesticide contamination of different bodies of superficial water (e.g., lakes, rivers) [85]. Moreover, these two sectors use approximately 87% of the total water used annually, and only about 25% of operation effluents are treated [85,86], thus increasing the risk of poorly treated water making it back into rivers and streams, and contributing both microbiological and chemical contaminants that can enter the food chain.

Only a few studies have looked into pesticide residues in water sources [87,88]. Metabolites of DDT were reported at concentrations of 0.010–0.12 µg/L in rivers in Tarapoto, Peru [87]. Another study detected traces of chlorpyrifos (0.69 ng/L) and DDT derivatives (0.0059 ng/L) in the water of Alto Mayo, Peru [88] The presence of DDT derivatives in both studies could be attributed to either illegal use or its low biodegradability since the time it was banned in the 1990s. On the other hand, the presence of chlorpyrifos, an approved organophosphate insecticide, may be linked to its frequent application during agricultural activities.

In contrast to the limited studies of pesticide residues in water, there are numerous works that report the presence of heavy metals in water, and these are summarized in Table 6. The most frequent elements in water sources with noncompliant values included As, Cd, and Pb. For example, the Rimac River was reported to have high concentrations of 0.27 mg/L in Pb and 0.016 mg/L in Cd, exceeding Peruvian MCL in irrigation water [89]. The Rimac River is important for the densely populated departments of Lima and Callao, as it serves many purposes including farming, mining, power, and drinking water. Another study reported that irrigation canals that sourced water from the Mantaro River in Junin showed a concentration of 0.18 mg/L As [90]. Similarly, the Cunas sub-basin, a tributary of the Mantaro River, has also been shown to have exceeding levels of As, at 0.60 mg/L [91]. Chemical contamination has been a problem in these two rivers since initial reports in 1986 [92]. Since agricultural crops are irrigated with water from these rivers, this indicates that crops in Junin and Lima are at risk of heavy-metal contamination. Although increasing sources of water is a growing necessity throughout Peru, so is ensuring access to higher-quality water.

## 6. Chemical Contaminants and Public Health

It is estimated that chemical contamination, from all sources including food, is responsible for 4.9 million deaths and 86 million disability-adjusted life years per annual basis around the world [100]. Chemical contaminants in foods have serious human health consequences, ranging from minor stomach problems to death. The specific health effects for heavy metals, mycotoxins, and pesticides have been described elsewhere [74,101,102]. These contaminants tend to be associated with a complex array of health manifestations based on dose, ranging from acute (e.g., nausea, vomiting, tremors) to more chronic (e.g., suppression of immunity, cancer, neuropathy, and organ damage) and lethal effects.

Reports linking pesticide residues as the causative agent of foodborne outbreaks in Peru were associated with acute intoxications. These poisonings entailed three massive foodborne outbreaks that involved a total of 255 individuals and led to 36 deaths. The first outbreak occurred in 1999 in Tauccamarca, Cusco, where 50 children in a school were intoxicated after consuming a powdered-milk substitute tainted with parathion [103]. This exposure led to 24 deaths, while the remaining 26 survived and suffered from dizziness and convulsions [104]. In 2011, 97 children were poisoned, three of whom died after eating lentils and rice contaminated with carbofuran at a school in Cachachi, Cajamarca [103]. Another outbreak involving parathion occurred in 2018 at Paucar del Sara Sara, Ayacucho, affecting 111 people, and killing 9 of those who ate mote soup at a wake [105]. It is alarming that two of these outbreaks involved parathion, an extremely hazardous (or Class Ia according to the WHO) organophosphate banned from use in Peru since 1998. The parathion tragedies were 20 years apart, yet the most recent outbreak emphasized that law enforcement regarding this pesticide has not been adequate and requires attention. This growing necessity for law enforcement is especially true, as parathion and other forbidden pesticides can still be purchased without a technical prescription in Peru [104].

Another type of pesticide intoxication indirectly related to produce food safety is that of occupational poisoning. The Ministry of Health (Ministerio de Salud, MINSA) reported that more than 2000 cases of acute poisoning (symptoms including headaches, temporary loss of vision, loss of consciousness, and dizziness) have occurred annually since 2016 [106]. It is likely that such poisonings were due to pesticide applicators not following the instructions on the labels of pesticide bottles. This hypothesis is supported by multiple Peruvian surveys, which showed that at least 50% of intoxications were due to mishandling of methamidophos, a highly hazardous organophosphate insecticide [107,108,109]. These intoxications occurred when applicators either failed to read the instructions or did not have the appropriate protective equipment to apply pesticides. Following the label is critical, as it relays information on what to wear when applying pesticides, as well as on the frequency and dosing of application, which are key to not exceed the MRLs as described above.

**Figure 2:**
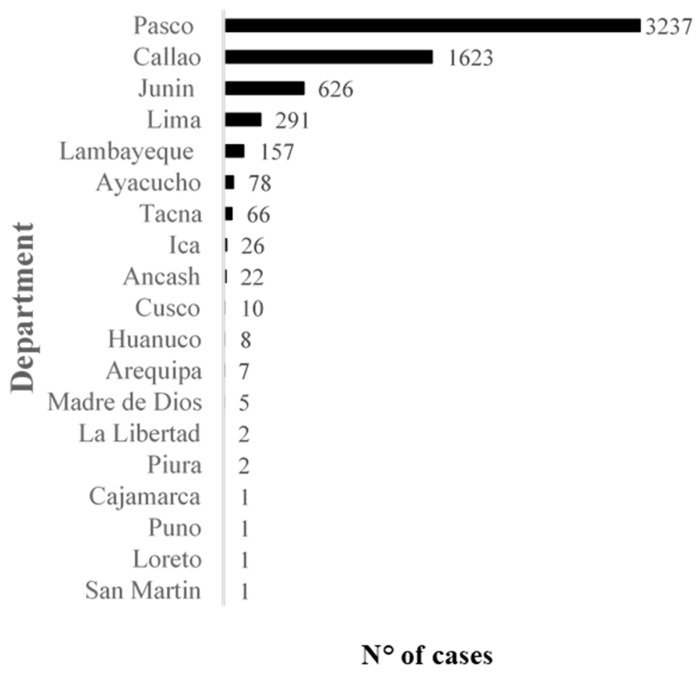
Number of acute heavy-metal-related intoxications in Peru in 2018. Data adapted from a report by the Ministry of Health (MINSA, 2019b).

Acute heavy-metal intoxications have also been reported, but from all sources and not only from food. It was recorded that more than 6000 people were poisoned by heavy metals throughout Peru in 2018 (Figure 2). As discussed earlier, heavy metals are problematic in Junin and Lima, but also in Pasco and Callao, accounting for more than 96% of the total cases per year [110]. One study agreed with these trends, as it evaluated the Pb blood levels of pregnant women from the metallurgic city of La Oroya, Junin [111]. It was found that maternal blood Pb levels from 24/30 (80%) participants were above 10 µg/dL, putting mothers at a higher risk of spontaneous abortion [112,113]. Another study of 346 children from the same city found that only about 0.9% of the children had blood Pb levels under the recommended 10 µg/dL, but children with exceeding levels of Pb did not receive continued monitoring [114]. This was in line with the MINSA report, which showed that children account for more than 80% of the cases of acute toxicity [106].

Lead toxicity has a long history as a critical issue in Peru, granting it the status of the leading country with the highest number of Pb intoxications in children in the Americas. Approximately 867,968 children (~10%) have blood Pb levels above 10 µg/dL, the Peruvian reference value, and 7,132,941 children (84%) have blood Pb levels above 5 µg/dL, the US Centers for Disease Control reference value [115]. Blood Pb levels above 10 µg/dL have been previously demonstrated to lower cognitive abilities in children of ages 6 to 9 in Callao [116], thus suggesting that Pb exposure can affect areas of the brain and contribute to cognitive decline. Furthermore, as the values of blood Pb levels go up, so does the risk of death. This was clearly shown in a study from Chile, which detailed the case of a girl with a blood Pb concentration at 104 µg/dL [117]. Consequently, the girl died from symptoms related to severe anemia. This case aligned with severe Pb toxicity, described as when blood Pb levels are higher than 70 µg/dL [118]. The Chilean case motivated investigations that pinpointed consumption of wheat flour that was contaminated with Pb during milling as the source of the issue, and the batch of wheat flour was destroyed. This highlights the importance of appropriate follow-up in medical cases that could help prevent serious health issues, such as that of dietary Pb intoxication.

Reports of mycotoxin toxicity in Peru are also very few in number, and the available data is suggestive of a possible association. The two studies of consumption of red chili peppers determined that these products were contaminated with AF and OT A, and interestingly, the Peruvian population has a high incidence of gall bladder cancer [76,77]. Similar results were observed in Bolivia and Chile, two high consumers of this pepper variety [76,119]. Since mycotoxins are associated with many types of cancers, it is no surprise that consumption of these fungi metabolites is being considered as a risk factor for gall bladder cancer [120,121]. The findings encourage the need to control the conditions to avoid the burden of mycotoxin production, and potentially reduce the risk of cancer.

While studies linking cancers and chemical contaminants are limited to mycotoxins in Peru, heavy metals and pesticide residues do pose a carcinogenic risk [122]. Because of the presence of these chemicals at noncompliant levels in Peruvian produce, it is prudent to assume the consumption of these contaminated foods as a risk for the development of cancer. Approximately 19% of all deaths were attributed to cancers in 2003–2016 in Peru, with stomach cancer being a main contributor to the mortality rate [123]. This statistic is suggestive of a plausible risk of chemically contaminated produce to human health. Moreover, it has been estimated that 70% of cancer-related deaths will come from low- and middle-income economies by 2030, which would be an increase from the current 56% [124]. Thus, decreasing the level of chemical contaminants in foods in the upcoming years is a crucial necessity in Peru.

## 7. Opportunities to Reduce Chemical Hazards in Produce

This comprehensive review identified that chemical contaminants in Peruvian produce have frequently been reported to exceed the recommended MRL, becoming a food-safety problem. There is a dire need for closer monitoring for attributing a specific source (e.g., produce or other foods) to chemical intoxications in Peru. The presence of chemical contaminants in sources (e.g., irrigation water) that are directly related to agriculture practices increases the vulnerability of farmed crops. Produce can be chemically contaminated throughout the multiple phases of the farm-to-fork cycle, including farming in the field, during pre- and postharvest activities, storage, packaging, transportation and distribution, and the consumer. Although the laws and regulations regarding chemical contaminants in foods do exist in Peru, the available reports and studies reflect that the current legislation enforcement is not sufficient for appropriately regulating these contaminants in fruits and vegetables.

Crop-management practices can impact the degree of chemical contamination of produce. One key problem in Peruvian agriculture is the reduced awareness about control points to decrease the occurrence of these contaminants in foods. The excessive application of pesticides and the common absence of personal protective equipment during pesticide application are examples of malpractices that can result in chemical contamination affecting produce consumers and field workers, respectively. Pesticide applicators should have a good understanding of how to handle, store, and dispose of these chemicals. Applicators should inspect and calibrate the pesticide-application equipment before spraying crops. Calibration is important to ensure that the pesticide is applied at the right rate over a target area to protect the crops, and reduce nontarget spray damage. The EU established a guideline that can be used as a reference for developing a plan for sustainable use of pesticides [125]. Implementing these measures can help reduce the impact of pesticides on human health and the environment.

Training growers is essential to attain a safer cultivation of foods in Peru. The SENASA agency began to offer trainings on pesticide application in 2015, and has since reached out to more than 10,000 growers in Peru [126]. Other trainings for growers in Peru have been focused on practices to reduce mycotoxin load on grains, with the prior approval of SENASA [127]. Such initiatives are appropriate to increase food-safety awareness, and include capacity-building for a better understanding of good agricultural practices, integrated pest management, and mycotoxin-prevention methods. It is important that these trainings are inclusive of growers in rural Peru.

Using untreated water for crop irrigation from rivers that carry industrial effluents may influence pollutant levels in produce. However, farmers in rural areas have limited options for water sources. Thus, it is increasingly important to advocate and push for more compliant wastewater treatment practices. For example, low-cost materials of biological origin (e.g., biochar and agricultural byproducts) have been shown as adsorbents that effectively remove pesticides and heavy metals in water, and could be explored as remediation options [128,129,130].

It is difficult to assess long-term effects from chemical contaminants in food because chemical contaminants can come from multiple sources throughout a person’s lifetime, but consumers have opportunities to reduce the load of chemicals in their produce. Practices such as cooking, peeling, and washing may reduce the levels of pesticide residues in fruits and vegetables [131]. Peeling is the most effective method of reducing pesticide residues, since most pesticides are applied directly to crops. This includes the removal of various organochloride and organophosphate insecticides from potatoes [132]. The boiling method has also been demonstrated to reduce levels of chlorpyrifos residues by at least 50% in leafy greens and tomatoes [133]. While this is not an absolute solution to the pesticide-residue problem in Peru, implementation of these methods can be a cost-effective solution for consumers that worry about reducing the load of pesticides.

Growers have a vital responsibility to reduce chemical contaminants in produce, and should therefore become aware about the associated food-safety issues. An increased knowledge and understanding of food safety can in turn lead to changes in farm practices in Peru. Food safety in Peru requires a transdisciplinary problem-solving approach, involving the collaboration of the government, nongovernmental organizations, academic institutions, food industries, healthcare systems, and stakeholders. Forming partnerships with universities that emphasize agricultural extension as part of the mission is also an important step to tackle the challenges affecting the agricultural sector in Peru.

The following are recommended initiatives that could help reduce the chemical contamination in produce grown in Peru:

- Strengthen the education pipeline: academic partnerships could help develop and lead training programs that combine research-based knowledge and practical agricultural experience to disseminate information on food-safety practices to the food producers. Training opportunities should be designed and promoted in ways that are accessible to and that engage farmers to adopt and adapt practices that support both agriculture sustainability and food safety.
- Supportive regional agricultural services: investment from the public and private sectors in infrastructure to support small-scale farmers with facilities and equipment, such as refrigerators for storage, and regular calibrations and technical checks of pesticide sprayers for more precise and effective pesticide application and protection of crops.
- Research partnerships: promote research that would develop methods to establish baseline levels for chemical contaminants in soils, irrigation water, and fruits and vegetables. Such baseline data can help locate soil fields, water sources, and crops that are at higher risk of chemical contamination and that require corrective interventions, thus promoting a stronger connection between the food industry and public health.
- Harmonizing food systems and public health: increased coordination and cooperation with the Peruvian healthcare system to implement surveillance systems that will be able to conduct traceback investigations to identify sources of disease outbreaks and stop current foodborne illnesses. Additionally, effective surveillance systems should also be able to estimate the long-term burden of foodborne diseases, evaluate priorities, and devise control and preventive measures.

## 8. Conclusions

Exposure to dietary chemical contaminants is well known to have a negative effect on human health. The existing literature highlights that chemical contaminants in Peruvian produce are an issue that deserves continuous monitoring and a thorough health-risk assessment. Otherwise, dietary exposure to these contaminants will increase the risk of chronic diseases, including cancer. Common activities like abundant mining operations and inadequate agricultural practices are challenges for both food safety and public health. A better understanding of the source of chemical contaminants and implementing management practices that can reduce food contamination and human exposure are needed. Addressing these challenges should go hand in hand with improvements in capacity-building and promoting collaboration. Thus, the government, universities, nongovernmental organizations, and the public and private sectors need to come together and invest more efforts to create awareness of the contaminants around them and the importance of management practices that prevent food contamination. Growing safer foods will ultimately benefit both public health and economic prosperity in Peru.

## Figures and Tables

**Table 1:** Heavy-metal quality standards for agricultural water and produce in Peru. Data adapted from the Food and Agicultural Organization and the World Health Organization (FAO/WHO), and the Ministry of the Environment [12,17].

Heavy Metal	Maximum Contaminant Limits				
	Irrigation Water (mg/L)		Fruits (mg/kg)	Vegetables (mg/kg)	Grains (mg/kg)
	WHO Standards	Peru Standards			
As	0.1	0.1	NE	0.1	0.2
Cd	0.01	0.01	0.2	0.1, except for	0.1 in cereals
				0.2 in leafy greens	0.2 in rice
					and wheat
Pb	5	0.05	0.1	0.1, except for 0.3 in leafy greens, and 0.2 in pulses	0.2
Cr	0.1	0.1	NE *		
Mn	0.2	0.2	5 *		
Zn	2	2	100 *		
Cu	0.2	0.2	30 *		
Hg	NE	0.001	0.02 *		

NE, not established. * Value designated for fruits, vegetables, and crops.

**Table 2:** Percentage of pesticide use by every 100 farms in each region of Peru.

Region	Chemical Insecticides		Herbicides		Fungicides	
	Farms that Apply	Farms that Do Not Apply	Farms that Apply	Farms that Do Not Apply	Farms that Apply	Farms that Do Not Apply
Coast	67	33	55	45	52	48
Sierra	37	63	14	86	25	75
Amazon	16	84	29	71	14	86

**Table 3:** Levels of pesticide residues in Peruvian produce. Adapted from multiple studies.

Produce	Pesticide Residue	Mean Concentration (mg/kg)	MRL ^1^	Reference
Potatoes	Methamidophos	2.7	0.05	[27]
Mandarin	Thiabendazole	5.1	5	[30]
Tomato	Methamidophos	1	1	[28]
Peas	Fenhexamid	0.16	0.05	[30]
Table grapes	Methomyl	0.12	0.05	

^1^ Maximum residue limit (MRL). Values according to the National Service of Agrarian Health agency [29].

**Table 4:** Chemical compliance of samples of plant origin monitored from 2011 to 2019 in Peru. Table adapted from annual reports by the National Service of Agrarian Health agency [31,32,33,34,35,36,37,38,39].

Year	Total	N° of Noncompliant Samples (%)		Departments with >40% Noncompliant Samples
		Pesticide Residues	Mycotoxins ^1^	
2011	252	73 (34.5)	14/42 (33.3)	Cajamarca, Ica, Piura, Tacna.
2012	725	193 (26.7)	8/62 (12.9)	Tacna, La Libertad
2013	705	175 (24.8)	19/74 (25.7)	La Libertad, Tacna
2014	756	228 (30.2)	6/84 (7.14)	Arequipa
2015	733	216 (29.5)	11/84 (13.1)	Piura
2016	738	199 (27.0)	2/43 (4.65)	La Libertad
2017	761	76 (10.0)	6/77 (7.79)	None
2018	793	105 (13.2)	2/79 (2.53)	None
2019	1779	373 (21) ^2^		Amazonas, Arequipa, Apurimac, Ayacucho, Junin, Ica, La Libertad, Lambayeque, Lima, Piura, Tacna

Varieties of produce sampled annually included Brazil nut, paprika, olive, grape, tomato, avocado, mango, orange, mandarin, lemon, asparagus, onion, coffee bean, banana, artichoke, and lima bean. ^1^ Mycotoxin analysis assessed total aflatoxins (A1, A2, G1, G2) and ochratoxin A, only in paprika and Brazil nuts. ^2^ No distinction was made, and number includes high levels of pesticide residues, mycotoxins, and heavy metals (arsenic, cadmium, and lead).

**Table 5:** Heavy-metal contamination in produce grown in Peru.

Produce	Mean Concentration (mg/kg)						Origin	Reference
	Pb	Zn	Cu	Cd	As	Cr		
Apple	0.10	2.4	0.6	BDL	0.05	NM	Lima	[56]
Avocado	0.18 ^a^	2.9	0.32	BDL	BDL	NM	Morropon, Chulucanas	[54]
Barley	BDL	60	19	NM	0.14	NM	Junin	[57]
Cabbage	9.3 ^a^	1.1	BDL	BDL	BDL	NM	Trujillo	[54]
Cacao bean	NM	NM	NM	2.5	NM	NM	Huanuco	[58]
	2.2 ^a^	52	30	0.97	NM	4.83	Amazonas	[59]
	1.0	44	26	0.77	NM	1.0	Cajamarca	
	1.0	40	19	0.17	NM	BDL	Cuzco	
	1.5 ^a^	37	25	0.64	NM	1.0	Huanuco	
	2.7 ^a^	47	28	0.41	NM	BDL	Junin	
	3.8 ^a^	59	27	1.6	NM	1.0	Piura	
	1.7 ^a^	44	27	0.79	NM	1.0	San Martin	
	2.8 ^a^	52	26	1.8	NM	1.0	Tumbes	
	1.7 ^a^	74	NM	0.96	NM	NM	Ucayali, Huanuco	[60]
Carrot	17 ^a^	6.6	BDL	BDL	BDL	NM	Trujillo	[54]
Cassava root	5.8 ^a^	NM	NM	0.039	3.0 ^a^	NM	Hualgayoc	[61]
	0.13 ^a^	NM	NM	0.05	NM	NM	Junin	[62]
Cauliflower	4.4 ^a^	2.4	BDL	BDL	BDL	NM	Trujillo	[54]
Chili pepper	11 ^a^	3.0	0.32	BDL	BDL	NM	Trujillo	[54]
Cilantro	3.2 ^a^	4.0	0.89	BDL	2.050 ^a^	NM	Trujillo	[54]
	0.050	NM	NM	0.025	0.05	0.031	Arequipa	[63]
Corn	BDL	37	2.1	NM	0.0785	NM	Junin	[57]
Green pea	2.0 ^a^	5.5	1.7	BDL	BDL	NM	Trujillo	[54]
Lettuce	4.2 ^a^	2.5	0.63	BDL	0.900 ^a^	NM	Trujillo	[54]
	2.1 ^a^	NM	NM	0.1	NM	NM	Sierra	[55]
	0.37 ^a^	NM	NM	0.066	NM	NM	Costa	
Lime	0.12 ^a^	1.5	BDL	0.065	BDL	NM	Trujillo	[56]
Mango	0.087	1.5	BDL	BDL	BDL	NM	Chulucanas	[56]
Corn	0.010	NM	NM	BDL	0.030	NM	Arequipa	[64]
Onion	1.5 ^a^	0.70	0.23	0.023	BDL	NM	Trujillo	[54]
	NM	NM	NM	0.060 ^a^	0.042	NM	Arequipa	[65]
	NM	NM	NM	0.040	0.040	NM	Trujillo, Huaral	[65]
Orange	0.10	2.4	BDL	BDL	BDL	NM	Sullana, Lima	[56]
Papaya	0.090	2.0	0.63	BDL	0.20	NM	Sullana	[56]
Plantain	0.090	1.0	BDL	BDL	BDL	NM	Chulucanas	[56]
Plum	0.20 ^a^	2.5	1.1	0.023	BDL	NM	Piura	[56]
Potato	0.10	NM	NM	0.029	0.00040	NM	Lima	[66]
	BDL	NM	NM	0.31 ^a^	NM	NM	Cajamarca	[67]
	0.41 ^a^	18	NM	0.042	0.13	NM	Junin	[68]
Quinoa	0.040	NM	NM	0.020	0.060	NM	Arequipa	[64]
Rice	0.086	13	4.0	0.33	>0.20 ^a^	0.151	Tumbes	[69]
	0.040	NM	NM	0.11	0.17	NM	Arequipa	[64]
Strawberry	0.20 ^a^	3.5	0.30	BDL	BDL	NM	Trujillo	[56]
Tomato	1.9 ^a^	1.4	1.1	0.065	BDL	NM	Trujillo	[54]
Turnip	5.3 ^a^	2.7	0.59	BDL	BDL	NM	Trujillo	[54]
Watermelon	0.090	0.70	BDL	BDL	BDL	NM	Trujillo	[56]

^a^ Denotes values exceeding the maximum contaminant limit for the specific heavy metal in the specific produce variety. BDL, below detection limit; NM, not measured.

**Table 6:** Irrigation water contaminated with heavy metals in Peru. Values shown are mean or ranges.

Source	Location	Heavy-Metal Levels	Reference
Tumbes River	Tumbes	0.016 mg/L As	[69]
Cunas sub-basin	Junin	0.60 mg/L As ^a^ 19 mg/L Mn ^a^ 0.023 mg/L Cd ^a^ 12 mg/L Zn ^a^	[91]
Aimaraes sub-basin	Junin	0.010 mg/L Cd 51 mg/L Fe 2.1 mg/L Mn ^a^	
(Irrigation canals of) Mantaro River	Junin	0.18 mg/L As ^a^	[90]
Rimac River	Lima	0.47 mg/L As ^a^ 0.029 mg/L Cd ^a^ 21 mg/L Mn ^a^	[93]
		0.27 mg/L Pb ^a^ 0.016 mg/L Cd ^a^ 0.38 mg/L Cu ^a^	[89]
Chillon River	Callao	0.15 mg/L Pb ^a^ 0.012 mg/L Cd ^a^ 0.31 mg/L Cu ^a^	[89]
Lurin River	Lima	0.076 mg/L Pb ^a^ 0.018 mg/L Cd ^a^ 0.18 mg/L Cu	[89]
Grande River	Cajamarca	0.25 mg/L Pb ^a^	[94]
Nanay River	Loreto	0.11 mg/L Pb ^a^	[95]
Ananea River	Puno	0.16 mg/L Cd ^a^ 6.3 mg/L As ^a^ 1.4 mg/L Pb ^a^ 0.99 mg/L Cr ^a^	[96]
Tambo River	Arequipa	0.20 mg/L As ^a^	[97]
Quilca River		0.11 mg/L Pb ^a^	
Camana River		0.050 mg/L Pb	
Huatanay River	Cusco	0.0012 mg/L Pb 0.0036 mg/L As 0.0010 mg/L Cr	[98]
Caychihue River	Madre de Dios	0.20 mg/L Pb ^a^ 0.20 mg/L As ^a^	[99]
Moche River	La Libertad	0.0050–0.91 mg/L Pb ^a^ 0.0010–0.11 mg/L A s^a^ 0.0010–0.20 mg/L Cd ^a^ 0.045–1.2 mg/L Cu ^a^	[92]

^a^ Denotes value exceeding the maximum contaminant limit for irrigation water in Peru.

## Supplementary Materials

The following are available online at https://www.mdpi.com/article/10.3390/foods10071461/s1, Table S1: List of banned pesticides in Peru. Information according to the National Service of Agrarian Health agency. Table S2: List of pesticides commonly used in Peru.
