# Supplementary material for: Occurrence of Chemical Contaminants in Peruvian Produce: A Food-Safety Perspective
Source: Foods. 2021 Jun 24;10(7):1461. doi: 10.3390/foods10071461 (PMC8307517; doi:10.3390/foods10071461)
Supplement: Supplementary file 1 [file foods-10-01461-s001.zip › foods-1269808-supplementary.pdf]

Oscar A. Galagarza, Alejandra Ramirez-Hernandez, Haley F. Oliver, Mariel V. Álvarez Rodríguez, María del Carmen Valdez Ortiz, Erika Pachari Vera, Yakelin Cereceda, Yemina K. Diaz-Valencia and Amanda J. Deering

Occurrence of chemical contaminants in Peruvian produce: a food safety perspective

Table S1

List of banned pesticides in Peru. Information according to the National Service of Agrarian Health agency [1].

Pesticide Classification	Chemical Group and Active Ingredient
Insecticide	Carbamate
	Aldicarb
	Organochloride
	Aldrin
	Dieldrin
	Endrin
	DDT
	HCH
	Camphechlor/Toxaphene
	Endosulfan
	Heptachlor
	1,2-Dichloroethane
	Chlorobenzilate
	Pentachlorophenol
	Chlordane
	Chlordimeform
	Lindane
	Mirex
	Organophosphate
	Diethyl parathion
	Methamidophos
	Methyl parathion
	Monocrotophos
	Phosphamidon
	Inorganic
	Lead arsenate
Herbicide	2,4,5-T
	Dinitro-*ortho*-cresol (DNOC)
	Dinoseb
	Ethylene dibromide
Fungicide	Binapacryl
	Captafol
	Hexachlorobenzene
	Organomercurial compounds
Rodenticide	Fluoroacetamide

Table S2

List of pesticides commonly used in Peru.

Pesticide Classification	Chemical Group and Active Ingredient	EIQ Values [1]	WHO Classification Level [2]	No. of Countries Where Banned [3]	Banned in Peru	Crop Use [4]
Insecticide	Carbamate					
	Carbaryl	22.73	II	35	No	Potato, corn, bean, cotton, sorghum
	Carbofuran	50.67	Ib	63	No	Tomato, potato, banana/plantain, pumpkin
	Organophosphate					
	Methamidophos	36.83	Ib	83	Yes	Potato, bean, rice, tomato
	Diazinon	44.03	II	32	No	Onion, strawberry, corn, pineapple, alfalfa
	Dimethoate	33.49	II	4	No	Onion, alfalfa, avocado, cotton, tomato
	Methyl Parathion	35.22	Ia	61	Yes	NA
	Fenitrothion	NA	II	28	No	Alfalfa, cotton, rice, citrics, orange, corn
	Chlorpyrifos	26.90	II	4	No	Tomato, onion, corn, asparagus, pea
	Disulfoton	101.8	Ia	37	No	Potato
	Monocrotophos	NA	Ib	112	Yes	NA
	Malathion	23.83	III	2	No	Alfalfa, onion, garlic, rice, mango
	Trichlorphon	20.17	II	52	No	Corn, sugar cane, potato, chirimoya, peach
	Prophenofos	59.53	II	29	No	Potato, onion, squash, corn
	Azinphos methyl	53.05	Ib	80	No	NA
	Pyrethroid					
	Cypermethrin	36.35	II	28	No	Alfalfa, potato, cotton, asparagus, tomato
	Deltamethrin	26.38	II	NA	No	Onion, cabbage, cauliflower, soy, tobacco
	Permethrin	29.33	II	29	No	Alfalfa, artichoke, asparagus, onion, tomato
	Lambda-Cyhalothrin *	47.22	II	28	No	Potato, cotton, corn, tomato
Herbicide	Paraquat	24.73	II	46	No	Artichoke, banana, cacao, coffee, sugar cane
	Diuron	26.47	III	1	No	Cotton, coffee, pineapple, citrus fruits, apple
	Butachlor	NA	III	31	No	Rice
Fungicide	Tebuconazole	40.33	II	1	No	Asparagus, potato, rice, apple, mango
	Trifloxystrobin	29.78	U	NA	No	Asparagus, peach, strawberry, celery, peppers

Benomyl	30.24	U	34	No	Celery, tomato, citrus fruits, rice, apple
Penconazole	NA	III	NA	No	Peppers, mango, watermelon, pea, pumpkin
Metiram	40.61	U	NA	No	Celery, potato, rice, onion, beans
Triadimenol	26.96	II	NA	No	Melons, barley, pear, wheat, tobacco
Propiconazole	31.63	II	28	No	Celery, barley, rice, banana/plantain, coffee
Copper sulfate	61.90	II	NA	No	Onion, melons, pepper, garlic

[1] Environmental impact quotient (EIQ). The higher the number, the greater the impact with regard to the environment or human health. Retrieved from New York State Integrated Pest Management program from Cornell University [2]. [2] Toxicity classes defined by the World Health Organization: Class Ia, extremely hazardous; Class Ib, highly hazardous; Class II, moderately hazardous; Class III, slightly hazardous; U, undefined. [3] According to the Pesticide Action Network International [3]. [4] Data according to the National Service of Agrarian Health agency [4]. * Organic pesticide. NA, information not available.

References

1. Servicio Nacional de Sanidad Agraria (SENASA). *Plaguicidas Agrícolas Restringidos y Prohibidos en el Perú*; SENASA: Lima, Peru, 2015. Available online: https://www.senasa.gob.pe/senasa/plaguicidas-restringidos-y-prohibidos-en-el-peru/ (accessed on 15 March 2021).
2. New York State Integrated Pest Management (NYS IPM). List of Pesticide Active Ingredient EIQ Values. Available online: https://nysipm.cornell.edu/sites/nysipm.cornell.edu/files/shared/documents/EIQ-values-May-2020.xlsx (accessed on 15 March 2021).
3. Pesticide Action Network International. PAN International Consolidated list of Banned Pesticides. Available online: http://pan-international.org/pan-international-consolidated-list-of-banned-pesticides/ (accessed on 15 March 2021).
4. Servicio Nacional de Sanidad Agraria (SENASA). Consultas del Registro de Plaguicidas. Available online: http://200.60.104.77/SIGIAWeb/sigia_consulta_producto.html (accessed on 15 March 2021).
